# Consciousness, decision making, and volition: freedom beyond chance and necessity

**DOI:** 10.1007/s12064-021-00346-6

**Published:** 2021-05-28

**Authors:** Hans Liljenström

**Affiliations:** Agora for Biosystems, SLU, P.O. Box 7032, SE-75007 Uppsala, Sweden

**Keywords:** Neurocomputational modeling, cognition, decision-making, consciousness, volition, free will

## Abstract

What is the role of consciousness in volition and decision-making? Are our actions fully determined by brain activity preceding our decisions to act, or can consciousness instead affect the brain activity leading to action? This has been much debated in philosophy, but also in science since the famous experiments by Libet in the 1980s, where the current most common interpretation is that conscious free will is an illusion. It seems that the brain knows, up to several seconds in advance what “you” decide to do. These studies have, however, been criticized, and alternative interpretations of the experiments can be given, some of which are discussed in this paper. In an attempt to elucidate the processes involved in decision-making (DM), as an essential part of volition, we have developed a computational model of relevant brain structures and their neurodynamics. While DM is a complex process, we have particularly focused on the amygdala and orbitofrontal cortex (OFC) for its emotional, and the lateral prefrontal cortex (LPFC) for its cognitive aspects. In this paper, we present a stochastic population model representing the neural information processing of DM. Simulation results seem to confirm the notion that if decisions have to be made fast, emotional processes and aspects dominate, while rational processes are more time consuming and may result in a delayed decision. Finally, some limitations of current science and computational modeling will be discussed, hinting at a future development of science, where consciousness and free will may add to chance and necessity as explanation for what happens in the world.

## Introduction

A crucial aspect of the human nature is the experience of being a conscious agent, in control of my actions in the world. We (normally) experience that we have a free will that is not—in principle—determined by the world around us, even if it could be constrained by it. We may think we can make conscious decisions and act out of free will, but that is not a guarantee that it actually exists. So, what can we know about consciousness and free will, what can science say about it?

The issue of free will has been much debated in science and philosophy, as well as in religion, where it often has normative implications. In science, it has been discussed since at least the eighteenth century, whether the world is deterministic or not, and hence whether there is any place for a free will. If the world is totally deterministic, everything that happens in the universe is determined by the natural laws (and boundary conditions). Also in an indeterministic world view based on randomness at (primarily) microscopic levels, there is a difficulty in seeing a place for a free will. Hence, the two alternative explanations that science can provide for events in the physical world, randomness and natural laws, or *chance and necessity*, as coined by the French biochemist and Nobel laureate Monod (1971), seem to exclude the possibility of a free will.

It has also been argued, that if free will exists at all, it is only for humans, and no other species. For us humans, it is linked with the notion of responsibility and moral, which possibly may extend way beyond our pure human relations. There are different views on what science says and can say about the human condition, but the dominant view is, perhaps, best expressed by the following quotation by Crick (1994):

The Astonishing Hypothesis is that ‘you’, your joys and your sorrows, your memories and your ambitions, your sense of personal identity and free will, are in fact no more than the behavior of a vast assembly of nerve cells and their associated molecules… You’re nothing but a pack of neurons.

Certainly, there are alternative views, here represented by a quotation by Ellis (2009):

All scientific experiments are based on purposeful activity and free will, enabling decisions based in abstract analysis that lies beyond the explanatory scope of physical science…..bottom-up, same-level, and top-down causation all occur at the same time, in concert, enabling the emergence of genuine complexity based in modular hierarchical systems.

During the past decades, there has been a debate in the literature regarding some experiments apparently showing that free will is just an illusion. It seems that the brain knows, at least 0.5 s in advance what “you” decide to do (Libet et al. 1982, 1983). Lately, this time window has been extended to incredible 10 s, based on new experiments (Soon et al. 2008; Haynes 2011). The common interpretations of these studies have, however, been criticized (see e.g., Mele 2009, 2019; van Inwagen 2017), and alternative interpretations of the experiments can be given. Below, we will go through some of the experiments and their interpretations.

This paper deals with various experiments, theories and models related to the problem of free will. The work is also related to a newly started international project, *The Neuroscience and Philosophy of Free Will* (www.neurophil-freewill.org), which attempts to dig deeper into the questions, also developing more advanced and realistic experiments than previously done. An important question to sort out is to what extent consciousness is involved in volition, i.e., in acts of (presumed) free will. Although not all agree, consciousness seems tightly linked to free will, so we will first discuss the problem of consciousness, before discussing in further detail how it might relate to decision-making and volition.

## Consciousness

There is no consensus of what consciousness is, but many scientists (and philosophers) consider it to be an emergent phenomenon, resulting from the activity of billions of interconnected neurons in our brains. As far as we know, humans are the only ones that can report being conscious, but it is likely that many other organisms also have consciousness to various degrees. The degree of consciousness presumably depends on the complexity and organization of the nervous system (or other material substrate). Human brains consist of about 10^11^ neurons with altogether about 10^14^ synapses, connecting those neurons in intricate and highly organized networks. No one knows how many neurons, or what kind of networks or neural activity that would be required to allow for (any level of) consciousness, but there are scientific methods of various kinds to try to find out (see e.g., Tononi and Koch 2015). Yet, three decades after it was considered a scientific problem (Crick and Koch 1990), science is still not able to say much about what consciousness is, or how we should relate it to the brain and its processes. To understand consciousness is one of the major problems in science, and many open questions remain. Here, we adopt a dynamic approach to the problem.

Consciousness has presumably evolved, in smaller or larger steps, with the evolution of the nervous system (Århem and Liljenström 1997). At present, we cannot know at what stage in evolution, with what animals the first signs of conscious cognitive processes appeared, but it can be argued that mammals, and probably birds, possess this quality. These animals are believed to have Gestalt perception of objects and are able to think in abstract symbols, to a lesser or higher degree (for example, a chimpanzee can do this to a much higher degree than a mouse). They can be assumed to have subjective experiences, although not necessarily be aware of themselves as individuals (Griffin 1992).

However, it has been argued that consciousness, in some primitive form, could exist in much lower organisms, like insects or even single-cell organisms. For example, Delbrück (1986), the father of molecular biology, considered a continuous evolution of consciousness from the very early life forms until humans:

*Perception*, i.e., reception and interpretation of signals from the environment, must be of high evolutionary antiquity, being a common attribute of all contemporary forms of life. Even in single-celled organisms, all three components of the perceptive process of higher forms of life are present: *stochastic* response, *deterministic* response, and *adaptive* response based on immediate past experience.

Not only phylogenetically, but also ontogenetically, consciousness could be considered to expand and develop over time, to reach different levels at different stages of development (Changeux 2008):

Explicit ‘*self-consciousness*’ develops in infants at the end of the second year, together with working and episodic memory and language; it is characterized by self-recognition in mirror tests and by the use of single arbitrary rules with knowledge of one’s own behavioral potential and self-other distinction; to some extent chimpanzees might reach this level…. ‘*Reflective consciousness*’, theory of mind and full conscious experience, with first-person ontology and explicit report, is unique to humans and develops following 3-5 years in children.

Cognitive processes are clearly multi-leveled, and in humans these would range from unconscious to highly conscious cognitive activities. All cognitive processes are clearly not conscious. On the contrary, most cognitive processes are unconscious, but at some degree of complexity and arousal some cognitive processes become conscious. Furthermore, it is apparent that consciousness appears at different levels of complexity. There are reasons to believe that full human consciousness shows more levels than that of any other species. For instance, there is no clear evidence that other species possess more than rudimentary forms of self-consciousness, i.e., consciousness/awareness of a self and the capability to reflect about it. The emergence of self-consciousness and human language must have constituted a major transition in evolution. It implies a critical, goal-directed thinking which has changed the world dramatically.

Consciousness appears central for higher cognitive functions, in that conscious cognition may provide prediction, expectation, volition, plans, goals, hopes, etc. beyond the immediate perception. It would have consequences for choosing mating partners, for securing good living conditions for offsprings, etc. Free will could possibly develop out of desires, and this should constitute a strong driving force in nature. Thus, these conscious functions can be involved in increasingly complex interactions with the environment, including other individuals, as an essential part of an evolutionary process.

The complex neurodynamics of cortical networks, primarily at a mesoscopic level, seem significant for cognitive functions and conscious activity. It has been related to perception, attention and associative memory, but also to volition and activity in the sensory and motor areas of the brain. Even though many details are still unknown, it is obvious that there is an interplay between the neurodynamics of the sensory and motor pathways. This will be more thoroughly discussed in the following sections.

## Intentionality and free will

Clearly, the development of our cognitive and conscious abilities depends on an appropriate interaction with the complex and changing environment, in which we are embedded. Our perceptions and actions develop and are refined to effectively deal with our world. For this process, *intentionality* is a useful concept.

There are different views on the temporal and causal relationship between intentions and decisions, and to what degree intentions are conscious (see e.g., Haggard 2008, 2019; Mele 2009, 2019; Block 2021), but the difference partly depends on the context. For example, experimental situations are usually quite different from ecological ones. Here, we adhere to the view that intentions, which may be unconscious or conscious to various degrees, in general precede decisions, which typically would be conscious. This view links closely to that of Freeman (1999), who based his view primarily on physiological and behavioral studies of animals.

The process of intentionality, according to Freeman (1999; see also a review of Freeman’s ideas in Liljenström 2018) comprises the operations of predicting, planning and learning actions. Intentions imply the creation and projection by the brain of alternative future states, desired or feared. Such hypotheses are constructed in attractor dynamics by extrapolation from past experience, and they serve to control choices and directions of actions in the present. Presumably, intentionality cannot be explained by the common understanding of linear causality. Instead, *circular causality* could better explain it, in terms of “action-perception cycles”, in which each perception concomitantly is the outcome of a preceding action and the condition for a following action.

Three properties of intentionality could be identified (Freeman 1995): directedness (toward a goal), unity, and wholeness, which all could be associated with three stages of intentional behavior:

- 1. In the first stage, a stimulus is categorized in primary sensory cortex, usually accompanied by an experience of emotion and value;
- 2. In the second stage, different sensory modalities are integrated into *gestalts*, which are assigned time and place in the life history of the individual;
- 3. In the third stage, there is a global coherence over the entire cerebral cortex, resulting in a decision to act (or not to act).

In principle, there could be many intentions, both distal and proximal (in time) within our brain–mind system, most of them unconscious, and possibly competing, but one (or a few) of them may lead to a decision to act. There could be different causes for the build up of intentions, for example genetic, or physiological, but sometimes also emotional and cognitive. Hence, intentions may originate in different parts of the brain, including the limbic system.

Decisions, on the other hand, are cognitive in nature and primarily associated with higher cortical systems/processes. Hence, many animals may have intentions to do this or that, but never really make a decision to do so. Humans, too, while still largely influenced by our limbic system, would have unconscious intentions, perhaps, leading to actions without decisions. Some of our intentions might be cognitively, and perhaps, also consciously formed in neocortex, e.g., in LPFC. In such a case, an intention could result from a decision (such as in experimental situations with spontaneous finger movements, as will be discussed below). Typically, though, an intention, distal or proximal, could last for a longer period of time, and would precede a decision, which is more or less instantaneous. (I may for a long time *intend* to go to Paris, but I *decide* to do it only when I get an opportunity to do so, and then I act upon that decision and buy a train ticket). In some cases, when immediate actions are called for by environmental conditions, intentions and decisions may be more or less simultaneous.

*Intention* can be viewed as a precursor to (free) will, as an “urge” or “desire” to act in a certain direction, to attain a certain goal. Voluntary movement, or more generally, behavior, is based on perception and past experience (memory), which are required for prediction of (inter)actions. *Attention* may provide information about the internal and external worlds, but intention guides our actions (Liljenström 2011).

Intention and attention are related to action and perception, respectively, involving both the sensory and motor hierarchies of the nervous system in the action-perception cycle. Both of these aspects are essential for exploring our external, as well as internal world. Exploration, through attention-perception and intention-action, should be fundamental to all animals, but the exploratory capacity is likely to increase with increasing complexity of the nervous system (Liljenström 2011, 2018).

While intention and attention may be present in many organisms, higher conscious cognition, and in particular the awareness and reflection of a self, seems to appear only with human beings, eventually resulting in the notion of a free will and responsibility for our actions. Even though most of us *experience* that we can make conscious decisions and act out of free will, it is not an evidence that it really exists. In our daily life, we do not think or feel that we are governed by neither chance nor deterministic laws of nature. Yet, the general view in science and philosophy, as mentioned above, is that free will is an illusion. This view is founded partly on the belief that the world is deterministic, at least at the macroscopic level of our daily life, while the microscopic world of atoms and molecules may be largely random.

The notion of free will as an illusion is also based on psychophysical experiments, which seem to show that a decision, e.g., to move a finger, is preceded by a specific brain activity up to several seconds before we become aware of “our” decision. This will be discussed at some detail in the next section, where we will refer to the notion of free will as *conscious will*, which appears more appropriate for the experimental situations described.

## Experiments related to conscious will

What is the role of conscious will in controlling human actions? The subjective feeling of agency is so immediate that we normally are convinced that our conscious will really controls our actions. Yet, there are a number of experiments that seem to indicate that consciousness is not causally connected to volition.

As mentioned above, some influencial work (Soon et al. 2018; Desmurget et al. 2018; Haynes 2011) appears to offer experimental evidence for the notion that conscious will is an illusion. The experimental results are taken as support for the dominating paradigm of materialism, which excludes any mental to neural (downward) causation. Empirical conclusions should, however, be critically examined—in particular, if they appear to support the currently dominating paradigm. The issue of conscious will is of profound importance for understanding the human condition, and a careful approach to the problem is therefore essential.

We consider *conscious will* as the conscious intention to act. While recognizing that the nature of consciousness is not understood, it can be assumed that there is a neural correlate of conscious will, and that conscious will can be probed experimentally through the reports of human experimental subjects. The hypothesis of *illusory conscious will*, ICW implies that the conscious will is not on the causal path to the willed action in wakeful behaving humans. Conscious will is then rather of the nature of a simulation that occurs as an appendix to the alleged causal chain from subconscious intention to motor action.

Identity materialism claims that the neural correlate of conscious experience is identical to the conscious experience, but this correlate could well be on the causal path to the willed action. Both illusory and causative conscious will are therefore consistent with identity materialism. The same results could, however, also be given a dualistic, or interactionistic, interpretation. Therefore, empirical support for ICW would not have the alleged philosophical impact, as many people seem to believe.

## Brief critical review of the experimental evidence

**Fig. 1:**
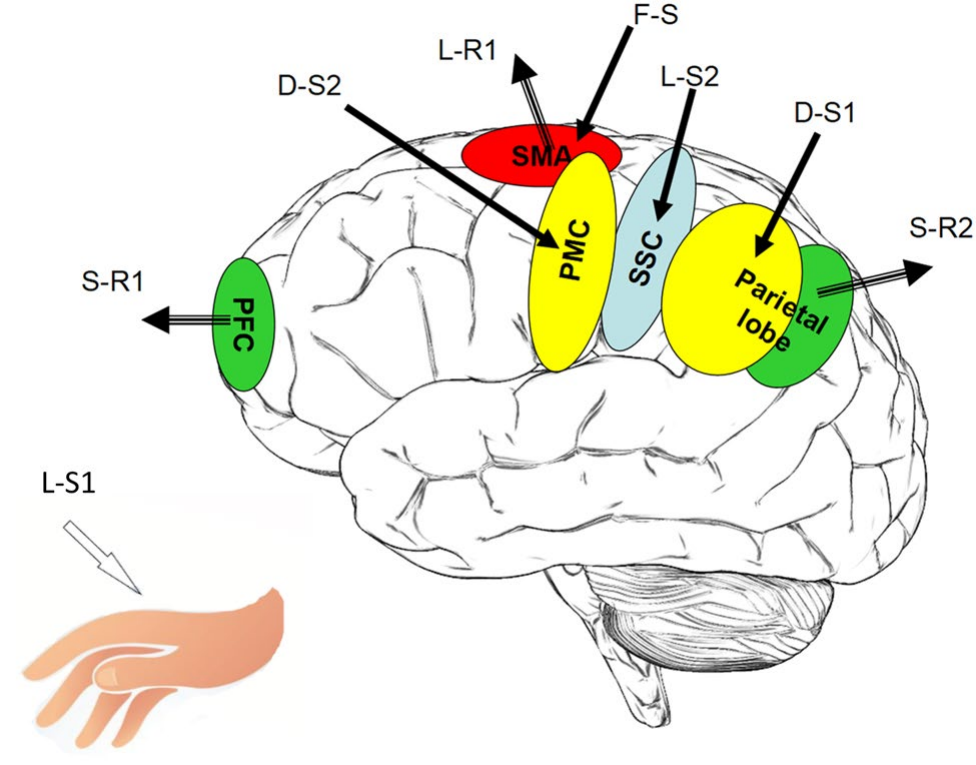
Schematic description of some of the experimental loci discussed in the text. SMA = Supplementary motor area, PFC = Prefrontal cortex, PMC = Premotor cortex, SSC = Somatosensory cortex. L-S1 and LS-2 corresponds to Libet’s stimulation of the hand, and of the somatosensory brain area, respectively. L-R1 is the recording of RP from SMA of Libet (and others). S-R1 and S-R2 correspond roughly to the brain areas, where Soon et al. detected nerve signals 10 s before the awareness of a willful act. D-S1 and D-S2 correspond roughly to the areas stimulated by Desmurget et al. F-S is the stimulation to the SMA by Fried et al.

In the following, we sum up the present evidence for ICW, contrasting its traditional interpretation with rarely considered alternative explanations of the data. Figure 1 summarizes the main experiments referred to in the text below.

### EEG evidence

A series of famous EEG experiments (Kornhuber and Deecke 1965; Libet et al. 1982; Libet et al. 1983; Keller and Heckhausen 1990; Haggard and Eimer 1999; see Libet (2004) for a review) are often quoted as evidence for ICW (See e.g., Paradiso et al. 2004, for similar results with implanted electrodes). The EEG *readiness potential* (RP), apparent only when averaged over a large number of trials, appears to precede the conscious will for spontaneous voluntary movements. Apparently, the conscious act of will is a part of an illusory time-delayed display. The conscious will is neatly edited into the mental video and makes us believe that we (as conscious subjects) are the masters of our behaviors.

The RP is found to begin about 1050 ms before the actual performance of the act in trials where subjects admit to be planning ahead, and about 550 ms when no such planning is reported. This means that the RP precedes the conscious will by about 850 ms with admitted pre-planning and by 350 ms without admitted pre-planning. This demonstrates, however, that the timing of the RP depends critically on pre-planning.

Little of the content of our stream of consciousness is available for verbal reporting, and subjects may often not recall or admit to pre-planned acts. The subjects are, however, well aware of the need to act within the next 10–100 s. Indeed, it is conceivable that the observed 350 ms effect is due to residual pre-planning and that the RP for genuinely unplanned actions is simultaneous with the subjective act of will. The earliest (5 out of 37) trials in Libet et al. (1983) averaged to be just 270 ms before the movement, hence being virtually simultaneous with the conscious will (see discussion in Trevena and Miller 2002).

### Evidence from brain imaging techniques

While EEG (and MEG) may elucidate temporal relationships in willed actions, at time scales of seconds, or below, brain imaging techniques such as PET and fMRI may provide better spatial information of which brain regions are involved, at time scales of several seconds to minutes. Studies by Frith et al (1991) point at prefrontal cortex as central in planning and choice of willed actions, but also SMA, parietal cortex and the basal ganglia seem to be involved (see e.g., Ingvar 1994; Spence and Frith 1999; Schultz 1999).

Soon et al. (2008) investigated timing correlations between subjective decisions and brain events measured by functional magnetic resonance imaging (fMRI). Subjects were asked to freely select between pressing either of two buttons operated by the left and the right hand, respectively. The subjects viewed simultaneously a stream of letters on a screen and the time for the subjective decision was measured by asking the subject to indicate the letter that was in focus when the decision was made.

While the subjects reportedly exercised their free will, fMRI scanning was applied to several brain regions including the supplementary motor area (SMA) and the frontopolar cortex. This technique measures changes in blood oxygen levels with high resolution and can hence assess the pattern of local brain activity. For some critical brain regions, it was possible to predict the handedness (left or right) of the action with at most about 60% accuracy, where 50% accuracy would indicate uninformed plain guessing. It was found that fMRI maps from the frontopolar cortex acquired 10 s before the action carried information leading to an on average 60% accurate prediction (see also Haynes 2011).

The second highlight of the Soon et al. experiments is that the timing of the action is predictable with 60% accuracy from fMRI data obtained from the SMA. The precise role of SMA is not known but it is typically assumed that SMA is involved in planning learned sequences of movements. The readiness potential that was detected in Libet’s experiments is generated in SMA.

Soon et al. concluded that “… the outcome of a decision can be encoded in brain activity of prefrontal and parietal cortex up to 10 s before it enters awareness.” Descriptions of the experiment have been published widely with the general slant of the comments that neuroscience finally has abolished free will by showing that real decisions are made subconsciously up to 10 s before the illusory conscious act of will.

However, a conservative interpretation of the same data is that there is a weak correlation between the brain state 10 s before the action and the action itself. This correlation could alternatively be understood as an effect of occasional unreported pre-planning, a correlation between the memory of the previous action and the next action, or a weak correlation between unconscious precursor processes and a causally connected conscious will.

### Brain stimulation evidence

Brute manipulation e.g., by open brain electrical stimulation (Delgrado 1969; Desmurget et al. 2009; Fried et al. 1991) or transcranial magnetic stimulation (Ammon and Gandevia 1990; Brasil-Neto et al. 1992) can induce a wide range of experiences, including conscious will (Haggard 2009). Such artificially enforced conscious will may occassionally be followed by appropriate motor actions or hallucinations of imaginary actions.

Electrical stimulation to the SMA can e.g., induce conscious will connected to real or imagined motor actions (Fried et al. 1991). For low intensity stimulation subjects reported an urge to move a specific part of the body. If the stimulation to the same point of the SMA increased the corresponding muscles actually contracted. This confirms other experiments and anatomical evidence indicating that the SMA is on the direct path from decision to action. The experiment indicates that a natural completed willed action is connected to a rising wave of potentiation in the appropriate part of the SMA. The urge is associated with the leading flank of the wave and is followed by the execution of motor actions triggered by the wave crest. This suggests that the urge to act functionally and temporally is associated with the real preparation for action. In this case, urge is not an illusionary delayed display of decisions that already have been made elsewhere (see e.g., Wegner 2003).

Desmurget et al. (2009) stimulate parietal and premotor cortex areas electrically during surgery in order to shed light on the role of these regions for conscious intention and motor awareness. They found that stimulation of parietal areas could trigger a strong intention and desire to move (a hand, an arm, a foot or the mouth), or if the stimulation was stronger, even could result in a feeling that a movement had been carried out. Stimulating the premotor areas caused mouth and contralateral limb movements, while the patients denied they had moved at all. The exceptional and artificial situation of open brain stimulation of brain areas related to planning triggers intermittently hallucinations of willed movement. However, this cannot be taken as conclusive evidence for ICW in normal wakeful subjects.

## Alternative hypotheses

In order to demonstrate the wide scope of interpretations of the experimental facts, we suggest an alternative hypothesis to ICW. The *causative conscious will* (CCW) hypothesis states that:

The earliest detectable sign of a decision in the human brain is never earlier than the subjective experience of the decision, as a conscious act of will.

The conscious will appears, according to the hypothesis, to be an active cause, since it always occurs before any willed action and no other cause is earlier than the subjective experience. The hypothesized timing of experienced will and causative brain events suggests that they are different aspects of the same thing. However, the physical or metaphysical nature of subjective will is not specified by the hypothesis. Conscious will could be a pattern of neural activation coherently coupled to conscious experience by a non-reductive psychophysical law, as suggested by Chalmers (1996). If so, one may speculate that conscious will in fact is connected to the key decision process in the brain. This would be consistent with the CCW hypothesis.

The CCW hypothesis applies only to situations where a conscious act of will occurs. Some decisions might be subconscious and bodily actions may in extreme cases be caused by electrical stimulation of relevant brain areas or a deep hypnotic suggestion (Wegner 2003). The CCW hypothesis states only that the conscious act of will, if it occurs, does not come after the physical decisions in the brain. Movement caused by open brain stimulation or hypnosis is usually not experienced as willful.

Typically, there are numerous external factors influencing, but not deterministically controlling, human actions. Such influences may well occur before the subjective act of will without violating the CCW hypothesis. There are also many influences within the brain that impact on later decisions. Such inputs to the decision-making process are, however, not equal to decisions.

Yet, in a complex system, such as the human brain, constantly interacting with its environment and with feedback loops within and without itself, it is difficult, if not impossible to determine any causal chains. We are in a continuous action–perception cycle. This is complicated by the different levels of organization of our nervous systems, where there are not only loops between different parts of the brain, but also between different levels (micro, meso, macro). We may not be able to say with certainty whether neural events precede mental events, or vice versa, or whether they are simultaneous. Simplified statements such as ICW or CCW can never fully reflect the complexity of the brain, but they may still be useful for analyzing what information we can gain from the experimental evidence. In the following, we will investigate whether data can falsify CCW.

### Conceptual causal pathways

The *neural correlate of conscious will* (NCW) is not directly causally connected to motor actions but is a part of the brain’s predictive simulation. There is an alternative model, where the neural correlate of conscious will is assumed to be a part of the planning funnel according to the CCW hypothesis.

Stimulating NCW in the open brain, as in the Desmurget et al. experiments, could result in a sensation of intention, although, due to the artificial nature of the stimulus, no appropriate signal is sent to the premotor areas and no action is triggered. This does not contradict the notion that NCW normally has a causal influence on actions. In contrast, stimulation of premotor areas triggers movements of mouth or limb, without an accompanying sense of intention, and even followed by a denial of movement. What these experiments apparently show is that the movements themselves are not sufficient for motor awareness. Rather, activity in the NCW seems necessary for such awareness, regardless if a real movement follows it, or not.

### The inner monolog interpretation

The traditional interpretation of experiments such as the ones of Libet and of Soon et al. rests on a highly simplified model of the stream of consciousness of the subjects. It is assumed that consciousness is blank, or at least uncorrelated to the experiment, until the subject suddenly experiences a conscious act of will. This model is critical for the interpretation of the experiments. The conclusion from the experiments would obviously be different if we discovered that the subjects had in fact (subconsciously) pre-planned all actions.

Soon et al. probed e.g., the awareness of the subjects at one specific occasion—the time of the conscious decision, approximately 1 s before the action. The consciousness of the subjects is, however, not empty when not probed by the experiment. The following hypothetical inner monolog may explain the observed events in the frontopolar cortex and the SMA, as correlates of a simultaneous conscious process.

**Table:** 

Time	Awareness	Observation
− 10 s	“OK, I have pressed right two times now—maybe it is time to make a left”	Frontopolar cortex signals a 60% inclination to press left
− 5 s	“I have waited almost long enough—it is soon time to press a button”	SMA signals a 60% correlation to pressing a button in 5 s
− 1	“I will press Left now”	The cortex signals the timing of the decision
0	“I am pressing Left”	The subject presses Left

Libet’s subjects were instructed not to pre-plan, but to “act spontaneously” within a given time frame. Subjects would have been censored by the researchers if they did not act at all for the duration of the experimental session, or if they kept bending the finger as fast as possible throughout the experiment. There was a clear expectation in the social context that the subjects should produce certain pre-specified “free will actions” with a certain mean frequency. The subjects may, in fact, consciously have intended to do so, but unconsciously pre-planned their actions.

Libet´s experiments demonstrate a strong dependency on pre-planning, and in fact the results from all experiments of this type are compromised by the presence of an unknown amount of pre-planning. Pre-planning distorts the result, so that it appears that conscious will is illusory.

### The memory correlation interpretation

A further problem in conscious will experiments is that subjects are asked to make voluntary decisions many times in a row, so that the scientists can compile enough statistics. This means that the subjects have memories of previous rounds in the experiment. Humans are not very good at random number generation. Most subjects would tend to select “Left” as the next free-willed action, if they already had chosen “Right” three times in a row. The next choice is therefore correlated with previous choices. When Soon et al. found a 60% correlation between the next decision and the state of the frontopolar cortex 10 s before the action, could that in fact be the correlation between the memory of previous choices and the upcoming decision? It is conceivable that the next choice in the series could have a 60% correlation with the accumulated history of the series.

### Multiple meanings interpretation

The experience of will is not clear-cut and is at best much weaker and more abstract than direct perceptual experiences. People can be genuinely uncertain whether they really have willed an action. Crime suspects can agonize over if a tragic incident was an accident or an intentional felony. This fuzziness is partly due to the lack of clear definitions of words like will, decision and intention, but it is also reflecting that the subjective act of will really is a wide spectrum of different mental states.

When subjects in the experiments by Jenkins and Ward (1965) erroneously thought that they wilfully controlled random external events, did they experience the same obvious act of will as subjects pushing a button in the experiment of Soon et al.? Or was it just a vague feeling of control of a subjectively different nature? Subjects are, in their reporting of conscious states, also strongly primed by the language used by scientists in the context of the experiments.

### The threshold stimuli interpretation

Libet’s experiments related to free will are, as we have seen, quite inconclusive as a stand-alone result. They are much more convincing as a part of the whole series of experiments that leads up to the hypothesis of backward referral of the conscious experience (see Libet (2004) for a review). The conclusions of the larger set of experiments seem to be that everything that we experience is like a time-delayed television broadcast of a sports event. Everything that we feel, do and want is a display of what the brain felt, did and wanted half a second ago. In that perspective, it is not surprising that the experienced will also is an illusory display.

However, Libet and others used threshold skin stimuli in the retroactive masking experiments. Sensory stimulation at intensity on the threshold to detectability might also require 500 ms to reach consciousness. If this process is interrupted by a suprathreshold signal, it will sometimes fail to register subjectively. This phenomenon is another artifact of subthreshold stimulation and must not be taken as evidence for subjective backward referral.

### The free veto interpretation

Libet thought that an RP may not be a sufficient condition for an act to occur, since an intended act, preceded by an RP, can be aborted by a veto signal. According to the “free veto hypothesis”, the conscious mind can, in extreme situations, override the initial impulse that is reflected in the RP. A person, who feels an urge to hit someone, also has the capacity to veto the subconsciously generated impulse. The free veto saves at least part of our moral responsibility. It is, however, easy to imagine a situation where the ethically correct behavior requires a positive action, and not just to suppress primitive urges.

### Re-considering the readiness potential

The RP has, ever since it was first measured by Kornhuber and Deecke (1965), been associated with a more or less conscious decision to make a movement (of a limb). The experience of this has been associated with a sense of acting out of free will. However, some recent experiments (Maoz et al. 2019; Mudrik et al. 2020) seem to indicate that the readiness potential found in Libet’s experiments with arbitrary choices is not found for more deliberate choices, where free will is more likely to come into play. This relates to the criticism that the kind of experiments by Libet and others do not test for free will at all, as there are no consequences of the arbitrary choice of movements made (e.g., moving a finger, pushing left or right button or similar). In addition, Schurger et al. (2012) suggest the RP reflects the average of accumulated stochastic fluctuations in neural activity, rather than a specific signal related to self-initiated action, although this hypothesis has recently been disputed (Travers et al. 2020). Still, the role of the RP in volition as well as the timing of different events in the Libet type experiments is debated (Schultze-Kraft et al. 2016; Lindahl and Århem 2019; Travers et al. 2020).

## A neurocomputational approach

Decision-making is, perhaps, the most important of our cognitive processes related to behavior, and is crucial for survival of all higher animals. In humans, conscious decision-making is linked to a sense of conscious/free will. We have used computational methods to address the problem of decision-making (DM), as an essential part of volition, and where consciousness supposedly plays a major role. Before describing our modeling, we will first give a brief introduction to the neural basis of DM, as we understand it.

### Decision making

Our actions are to a large extent a result of DM, which can be categorized into three phases: (1) Prevailing alternatives concerning the internal and external states are *emotionally* evaluated and prioritized, (2) a *cognitive* assessment of the options and the selection of actions, depending on needs (Damasio 1996). (3) The execution of an action, and evaluation of the resulting effect, which allows for a comparison between actual and expected value. Based on the “prediction error”, the assigned values to the choices in the first step are revisited and learnt, possibly resulting in a change of mind (Gold and Shadlen 2007; Doya 2008).

It is apparent that the DM process may not be as rational as often believed, which has been demonstrated by Kahneman et al. (2011). According to their hypothesis, DM is the result of an interplay between an intuitive/emotional and a rational/cognitive system, represented as *System 1* and *System 2*, respectively. This dual process model posits the integration of a “bottom-up”, intuitive, fast, implicit, emotional system (1) and a “top-down”, deliberative, slow, explicit, cognitive system (2). Below is a summary of the main steps of DM, according to the current theory.

Amygdala, as a part of the limbic system, has since long been associated with emotional processing, related to sensory perception and learning, linking the stimulus provoking emotional response and its emotional value. The functionality of the amygdala is realized through its connection to the orbitofrontal cortex (OFC), which receives extensive neural afferents from different sensory modalities. The bidirectional connections between these two structures are supposedly embodied in an affective DM process, where the perception and evaluation of environmental stimuli constitute the first phase (Barbas 2007).

In the second phase, the emotionally assessed stimuli in the amygdala-OFC pathway are monitored cognitively and a final decision could be taken. The lateral prefrontal cortex (LPFC) is considered to be mainly responsible for the cognitive evaluation of stimuli. In contrast to OFC, which pursues short-term rewards, LPFC primarily values future rewards. LPFC contributes to the prediction of the expected cognitive optional values. The resultant value of emotional and cognitive evaluations guides the action selection (Gray et al. 2002).

The outcome of the second phase is followed by the execution of an action. The real value of the selected action, as it is experienced, is evaluated and compared with the expected value by both OFC and LPFC. The computed prediction error provides the basis for learning. Through a recurrent pathway from LPFC to OFC a feedback signal is transmitted and the actual value of the performed action is maintained in OFC. Learning occurs in OFC by changing the neural firing frequencies, depending on the prediction error. The learning of new cue-outcome association and formation of adaptive behavior is also supported by OFC. The interconnection between amygdala and OFC associates the cue to its actual value and generates the outcome expectancy signal, which may induce a behavioral change (Dixon and Christoff 2014).

These systems (Systems 1 and 2) may correspond to “*fast* and *slow thinking”,* respectively (Kahneman 2011). Integration of the emotional and rational values in LPFC leads to the selection of one of several possible and available options. The output is transmitted to the motor cortex for the execution of an action.

### Model description

**Fig. 2:**
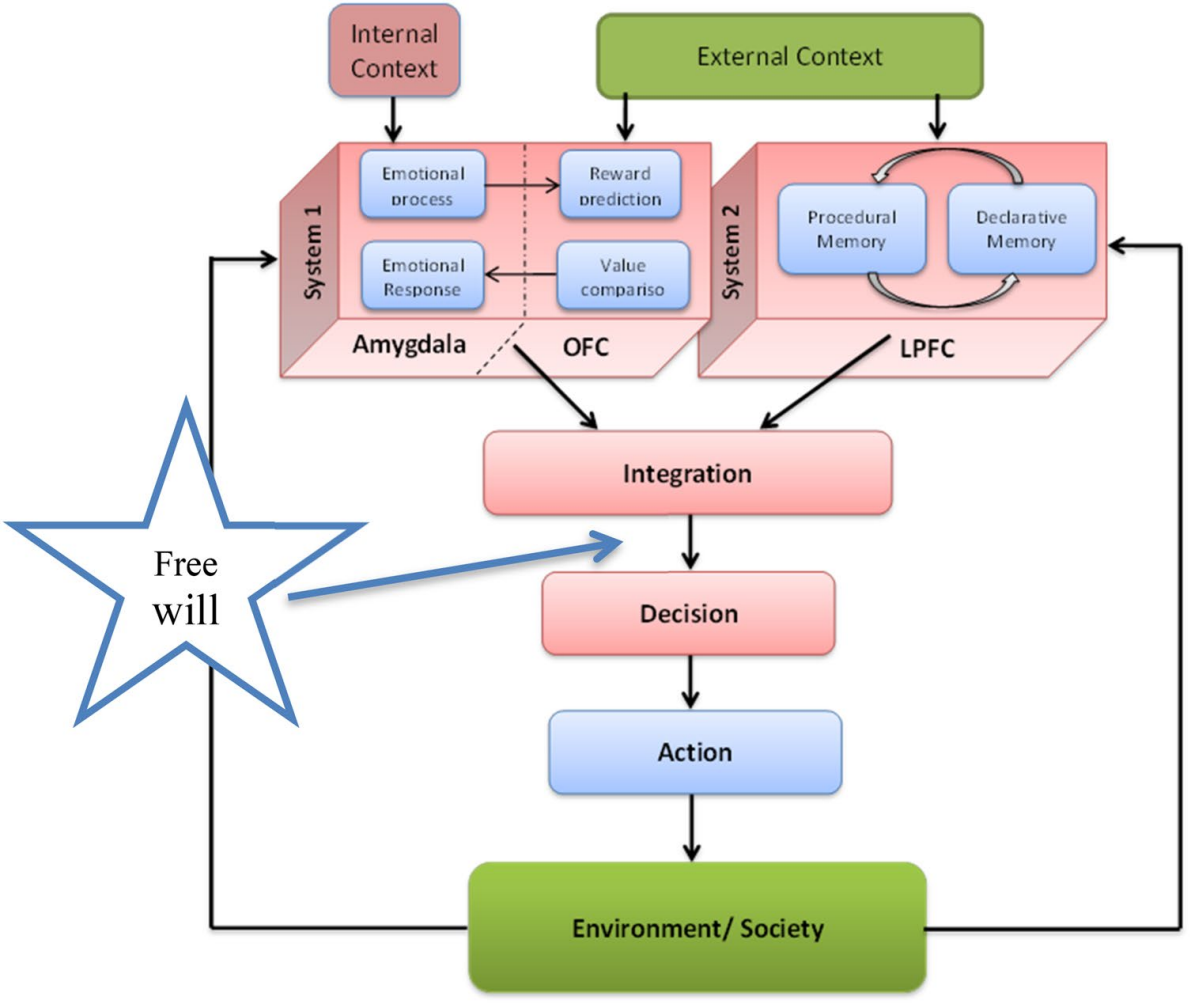
Our model of the different neural structures and processes involved in decision making, as based on the hypothesis that there is an integration of the signals from an emotional, fast system (System 1) and a cognitive, slow system (System 2). A possible “site” in the processing, where free will might be executed is indicated. (Modified from Hassannejad Nazir and Liljenström 2015)

As was outlined in the previous section, several key mental processes are involved when making a decision. The proposed model attempts to present an adaptive DM under varying internal and external contexts. Experimental results indicate the involvement of several different neural systems in the DM process, but to simplify the model, we focus our attention on three crucial neural structures: Amygdala, orbitofrontal cortex (OFC) and lateral prefrontal cortex (LPFC). The model encompasses an input-procedure-action-feedback process. This flexible process can be followed in both the emotional and rational/cognitive systems. The salient features of the two systems, *System 1* and *2*, are represented in our model, see Fig. 2.

The DM process is modeled with cortical neurodynamics at a mesoscopic level. The structures and dynamics of the three brain areas are modeled with attractor neural networks (see below). Oscillatory rhythms encode information related to perception, cognition and emotional associations, and are the result of interactions between excitatory and inhibitory neural populations. The network activity can be envisioned as local field potentials (LFP) or electroencephalogram (EEG) readouts.

**Fig. 3:**
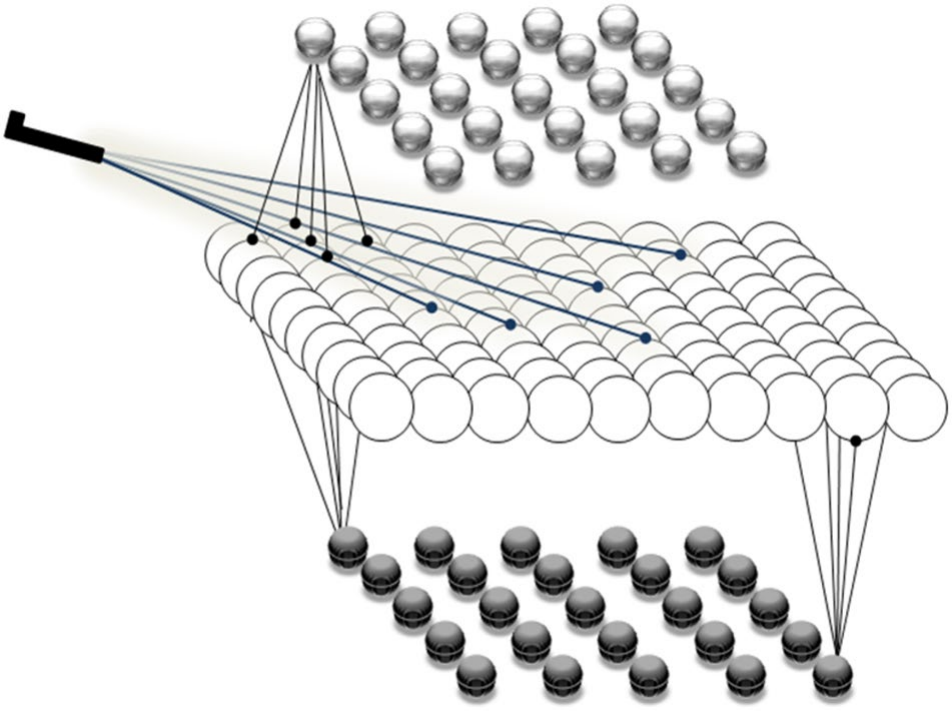
This figure represents three neural structures. The upper and lower layers are composed of 25 inhibitory neurons and the network in the middle is composed of 100 excitatory neurons. The external inputs stimulate (subset of) excitatory neural network. The stimulation of excitatory neurons is the start of activity of the system. Stimulated excitatory neurons excite inhibitory neurons which results in an excitation-inhibition balance

Our model is based on a previous developed cortical neural network model (Liljenström 1991), which has been extended and modified to mimic the structures of the amygdala, OFC and LPFC, respectively. The schematic network structure of the three systems is given in Fig. 3.

The excitatory sub-layer of each model structure consists of a network of 100 neural units (populations), while each of the two inhibitory networks is composed of 25 inhibitory units. The excitatory network nodes are connected recurrently, while there is no internal connection among inhibitory network nodes. An excitatory-inhibitory balance results from the bidirectional connectivity of excitatory units with the two inhibitory networks on either side.

Each unit in our model represents a group of neurons firing in synchrony. The oscillatory network behavior is a result of the interaction between excitatory and inhibitory neural groups, as described in more detail in Hassannejad Nazir and Liljenström 2015). The network structure allows for a complex neurodynamics, in particular oscillations with varying amplitudes and frequencies. The oscillatory properties are characteristic for each structure, where the activity of each neural unit can be regarded as the mean membrane potential of the population resulting in a graded, rather than spiking, neuronal output.

The time evolution for a network of *N* neural units is given by a set of coupled nonlinear first-order differential delay equations for all the *N* internal states, *u*. With external input, *I*_*i*_(*t*), characteristic time constant, *τ*_*i*_, and connection weight *w*_*ij*_ between units *i* and *j*, separated with a time delay *δ*_*ij*_, we have for each unit activity, *u*_*i*_, (with *x*(*t*) representing noise):

1 $$\frac{{{\text{d}}u_{i} }}{{{\text{d}}t}} = - \frac{{u_{i} }}{{\tau_{i} }} + \sum\limits_{j \ne i}^{N} {w_{ij} g_{j} [u_{j} (t - \delta_{ij} )]} + I_{i} (t) + \xi (t)$$

The input–output function, *g*_*i*_(*u*_*i*_), is a continuous asymmetric sigmoid function, experimentally determined by Freeman. This function shows the relation between input, as wave density, and output, as pulse density, in populations of neurons (Freeman 1979):

2 $$g_{i} = C \cdot Q_{i} \left\{ {1 - \exp \left[ { - \frac{{\exp (u_{i} ) - 1}}{{Q_{i} }}} \right]} \right\}$$

*C* is a normalization constant and the gain parameter *Q*_*i*_ determines the slope, shape and amplitude of the curve for unit *i*. This gain parameter is associated with the level of arousal or motivation, or alternatively, the level of a neuromodulator, such as acetylcholine. Thus, the curve nicely links the activity at the neuronal level with the activity at a behavioral level. Different values of the *Q* parameter can result in different dynamical states, going typically from static to oscillatory to highly irregular (“chaotic”) states, as the *Q* value increases.

The network connection weights *w*_*ij*_ are initially randomly set, but constrained by the general connectivity principles of cortical structures. To allow for learning, the weights are incrementally changed according to a learning rule of Hebbian type (Hebb 1949; Liljenström 1995):

3 $$\Delta w_{ij} = \eta g_{i} [u_{i} \left( t \right)]g_{j} [u_{j} (t{-}\delta_{ij} )](w_{\max } {-}w_{ij} ),$$

where the learning rate is denoted by *η*, and *w*_max_ is the maximum strength of an intrinsic synaptic connection.

External stimuli are driven by afferent neurons (not explicitly modeled) to a subset of the network of excitatory units. The difference between inputs to the emotional systems and rational one is modeled by different magnitudes. Excited units transmit signals to their excitatory neighbors, as well as to neighboring inhibitory neurons, which provide controlling inhibition to all excitatory neurons.

The function of the three neural structures requires the formation and update of cell assemblies. In our model, some specific parameters affect the oscillatory activities of the cell assemblies. A cell assembly with synchronized network oscillations, *s*, is the representation of stored experiences, attitudes and associated feelings toward the consequence of choosing any of the present options (alternatives) as the ultimate decision.

A decision in each of the emotional and rational systems separately is based on the “winning” response activity. The competition between the stored pattern toward the cue can be determined with the help of cosine similarity of the frequency and amplitude vectors ***f*** and *A*. The highest value of *V* will win the competition, and result in a decision to choose that option.

4 $$V_{{{\text{opt}}}} = |s| \times f \times A$$

The ultimate decision is the resultant of the emotional and rational decisions. The integration of emotion and cognition can then be computed by adding the emotional and rational/cognitive values, while considering the dominance of each structure over the other.

### Simulation example and results

In our model, some parameters are determined by the attitudes and priorities of an individual. Cell assemblies in amygdala, OFC and LPFC represent feelings, expectancy values, and rules for the outcome of a decision. As an example, we use the choice of travel from home to work, given there are three available optional means of travel. According to the three pillars of sustainable development, the rational and emotional attitudes considered are: (1) ecological (eco), (2) social (soc), and (3) economic/monetary (mon).

The choice depends on various external (weather, costs, traffic, etc.) and internal (mood, motivation, attitude, etc.) factors. Hence, we suggest there are three options to choose between: car (C), bike (B), and train (T), which all are considered to be available and reasonable (e.g., with regard to distance), albeit with different levels of convenience.

The value equation for e.g. car (C) can now be formulated based on the products of the probability (*P*_i_) and "utility" (*U(i)*) of choosing car, given its eco/soc/mon (*i*) values, where the "utility" is reflected by the oscillatory activity of the corresponding cell assembly:

5 $$V\left( C \right) = P_{{{\text{eco}}}} \times U\left( {{\text{eco}}} \right) + P_{{{\text{eco}}}} \times U\left( {{\text{soc}}} \right) + P_{{{\text{mon}}}} \times U\left( {{\text{mon}}} \right) = V_{{{\text{eco}}}} + V_{{{\text{soc}}}} + V_{{{\text{mon}}}}$$

If we assume that the decision maker is pro-ecological (climate) with her emotional and rational priorities regarding choice of travel as follows.

Emotional priorities: C > B > T; Rational priorities: B > T > C.

**Fig. 4:**
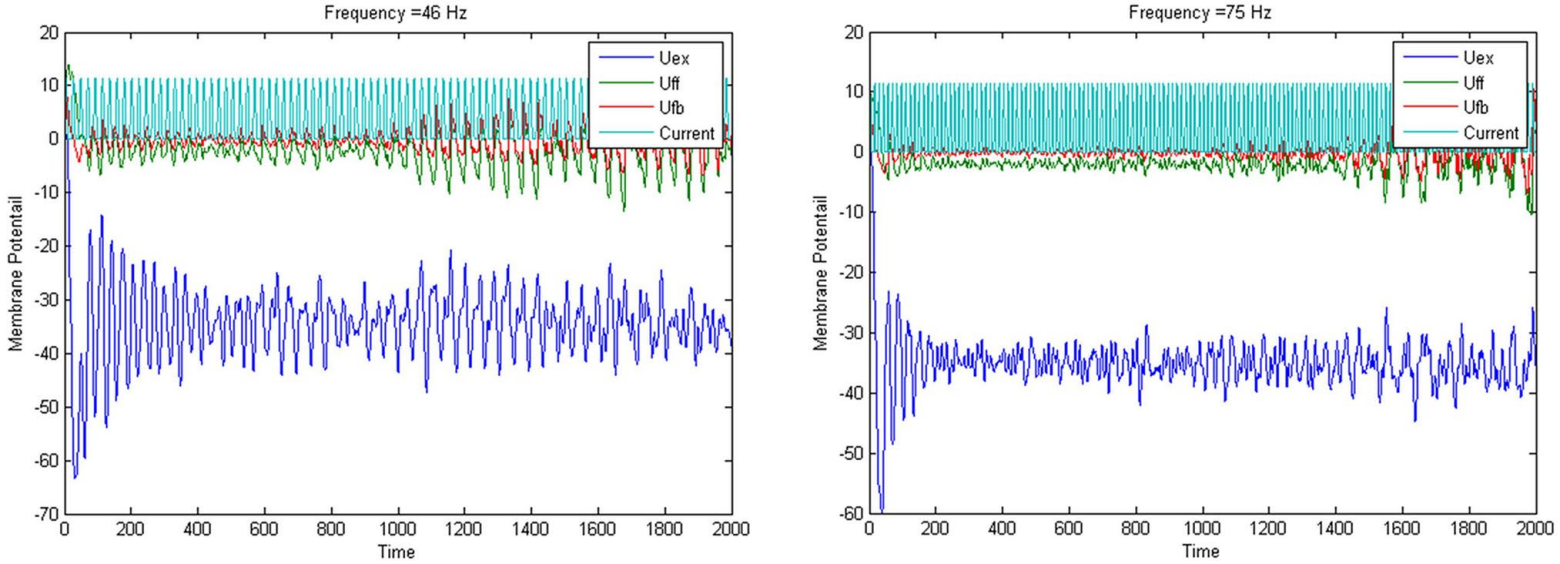
The oscillatory activity of LPFC cell assemblies representing the rational values for options "car" (*left*) and bike (*right*), respectively. The red and green traces show the mean membrane potential of the feedback (red) and feedforward (green) inhibitory network units, and the blue trace shows the oscillatory activity of excitatory units. The turquoise trace represents the input current to the networks. (Adopted from Hassannejad Nazir and Liljenström 2016).

In the absence of external/internal context, the priority should be followed and the option with the highest priority should be chosen in each system. In this case, C is the best choice emotionally, while rationally B is preferred. The cell assemblies that determine the association between the options and feelings and the consequence of taking an option are stimulated with external and internal stimuli. In the computer program the frequencies are just random number generated in the gamma range (30–80 Hz). As illustrated in Fig. 4, the randomly generated frequencies of emotional and rational memory, respectively, are both in the gamma range, but with different values. The difference between these frequencies is due to different external factors stimulating these two systems.

**Fig. 5:**
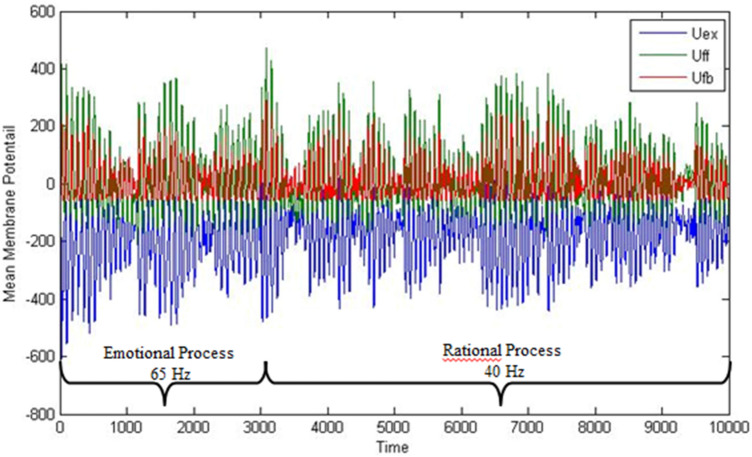
The oscillatory activity of assemblies in the emotional and rational systems representing the significance of choosing car as one of the options during the decision making process. In the simulation, the first 3000 ms represents the emotional processing and the following 7000 ms the cognitive/rational part. The higher frequency of neurons during emotional processing and lower frequency during rational processing indicate the higher emotional priority and lower rational priority of choosing car. (Adopted from Hassannejad Nazir and Liljenström 2016).

In Fig. 5, a simulation of the emotional-rational DM process is shown, where the first 3000 ms shows the emotional oscillatory activity and the last 7000 ms shows the rational analysis of the various options. The oscillatory activity is dominated by different frequencies (65 and 40 Hz) in the corresponding brain areas, *System 1* and *2*, respectively.

To summarize, with our neurocomputational model of DM, we have demonstrated how different cell assemblies, representing different optional choices available to an individual may compete with respect to their activity levels. The “winning” assembly is the one with the highest neural activity, measured as the product of the three assembly characteristics (number of cells, frequency, amplitude). Feedback from the environment of decisions/choices taken is also learnt and may influence future decisions. Preliminary simulations with our model indicate that if decisions are to be made fast, it is primarily emotionally related intentions which dominate, but if time allows for cognitive deliberation, the decisions may be more “rational”. Deliberate decisions are value driven, which is typical for conscious choices, in contrast to arbitrary decisions/choices. We are currently developing our model further, to include additional neural areas and processes, with the aim to explain and guide neurophysical and other kind of experiments with human subjects.

## Discussion and conclusion

In this paper, I have discussed some scientific research on consciousness and conscious (free) will, focusing on a series of neurophysical experiments, which have been used in the debate as evidence supporting free will as an illusion. In particular, I have discussed temporal aspects of willed actions and referred to experiments that attempt to associate the timing of neural events with such actions, and where subjects are able to report about their subjective experiences. Neurophysiological techniques, such as EEG are especially suited for this, as it has a high temporal resolution and is fairly easy to apply. These experiments are largely based on the findings that some “signal”, e.g., the so-called readiness potential (RP), precedes a conscious will to perform a movement.

I have further referred to brain imaging experiments, such as PET and fMRI, which are based on the relation between cortical blood flow and neuronal activity. These methods have a high spatial resolution, but a poor temporal resolution, and with the drawback of reflecting inhibitory activity, indistinguishable from excitatory activity. Brain imaging has revealed that a number of brain regions contribute to the performance of willed actions, in particular prefrontal cortex together with those brain regions with which it is connected (Spence and Frith 1999). Disease or dysfunction of these circuits may be associated with various disorders of volition, including “alien control”. I have also briefly described some experiments, where subjects are responding to electric or magnetic stimulation applied directly to the brain.

When carefully examining the experimental procedures and results discussed above, I can find no convincing evidence supporting the *illusory conscious will* (ICW) hypothesis. Similar conclusions have been made by, e.g., Heisenberg (2009), Sternberg (2010), Mele (2009, 2019) and Lindahl and Århem (2019). Further, recent studies by Schurger et al. (2012), Maoz et al. (2019) and Mudrik et al. (2020) even seem to undermine arguments for ICW based on previous interpretations of the Libet experiments. Batthyany (2009) not only questions the ICW hypothesis, but also points at the general bias in the interpretation of experiments, where the dominating philosophical preference abolishes alternative interpretations.

The alternative hypothesis of *causative conscious will* (CCW) is also neither falsified nor confirmed by the evidence. In fact, it is not an easy task to design and perform experiments that could reveal the true nature of willful acts, especially not in an artificial environment with non-ecological tasks. One could argue that experiments like those of Libet and Soon et al. do not test for free will at all, since the subjects are only asked to perform an “action” when there is an *urge* to move, and these movements can be said to be actions only in a very limited sense. Indeed, the way we pose our questions, set up our experiments, and instruct our subjects, is guided by our preconceived beliefs and assumptions, and hence it is difficult to get results that would contradict a dominating paradigm.

There is a great need for refined experiments and an unbiased analysis of the empirical evidence, which better can address the problem of conscious (free) will in natural complex situations. In particular since beliefs about our conscious will can have a significant impact on people’s moral outlook and behavior (Vohs and Schooler 2008; Sternberg 2010). The field also urgently needs a more precise and consistent terminology that avoids ambiguity and minimizes confusion (regarding concepts such as will, volition, intention, decision, and choice), although some attempts have already been made (e.g., Mele 2009). Such a terminology, if consensus about it could be reached, would facilitate interpretation and communication of hypotheses and experimental results.

In a newly started interdisciplinary project, *The Neurophilosophy of Free Will* (www.neurophil-freewill.org), we are actually attempting to sort out the various concepts related to consciousness and free will, as used in the literature. We also try to construct and carry out more ecological, realistic experiments, where initial results indicate that no RP appears for deliberate decisions in voluntary actions, as it does for arbitrary decisions (Maoz et al. 2019; Mudrik et al. 2020). In addition, we are using computational models that aim to suggest neural correlates and causative pathways included in decision making and volition.

In this paper, I have briefly described our initial computational modeling of decision making (DM), as an important part of volition. We have modeled various brain structures involved in DM, including the amygdala, the orbitofrontal cortex and the lateral prefrontal cortex, representing emotional, as well as cognitive aspects of the DM process. With our model we have demonstrated how different cell assemblies, representing different optional choices available to the individual may compete with respect to the level of activity. This level was suggested to be a combined measure of the size of the cell assembly (number of neural populations), and the frequency and amplitude of the oscillatory activity of the neural populations. The different options get different values, depending on internal as well as external factors. The “winning” assembly was simply the one with the largest neural activity, resulting in a decision, a choice for that option (with some probability).

A good model can help in understanding a system or process, predict the outcome of an experiment, or guide in new experimental studies. A model is, however, never correct, it is only more or less useful depending on the purpose. As long as the model output is interpretable in experimental results, or in a theoretical hypothesis, it may serve its purpose. Yet, when constructing a computational model of any neural system or process, we have to make tremendous simplifications. The problem is to find an adequate simplification, where the essential details are included, and the less relevant ones neglected. Indeed, one of the greatest challenges in the modeling process is to extract the relevant details out of the enormous amount of known facts about brain structures and their dynamics. Just simulating a neural process, or a cognitive function can never give any conclusive understanding of the system or process being modeled, just suggesting which solutions seem most plausible.

In our computational model, as in reality, there are many internal and external factors that all the time influence the DM process. In the complex neuronal networks of the brain, with many parts that interact and influence each other in myriads of feedforward and feedback loops, it is very difficult to determine any causal pathways, or what can be considered the first “independent” signal initiating the process leading to a decision, and subsequently to an action. Yet, neurocomputational models, such as ours, can serve as helpful tools when trying to describe and understand the underlying neural processes involved in DM, as a central part of volition and the exercise of free will. (In our further modeling, we also try to include effects of social interactions, which often are neglected in studies related to free will).

Decisions are based on an integrated evaluation of different emotional and cognitive assessment of the consequences of the decisions/actions, but it is not certain that a decision follows automatically the valuation made by the system. In our model, a decision is taken only with a certain probability, which is given by a random generator. In reality, it could be our more or less free will which allows for a possibility that we (as conscious subjects, see below) do not have to slavishly follow what the brain has calculated as the best decision in each case, which in general could be considered the most rational.

Undoubtedly, our decisions depend on, but are not determined by biological (genetic, neural, physiological) factors. They also depend on psychological, social and environmental factors, which altogether constitute a complex web of causation. This makes it hard or even impossible to predict an action or behaviour for any given individual, when studied “from the outside.” The apparent unpredictable outcome could be interpreted as a result of random neural activity, exemplifying upward causation, or as a result of free will, if downward causation is considered.

While there are no convincing scientific arguments for either ICW or CCW, based on experiemental evidence, it appears that ICW is easier to defend from a scientific point of view, because it seems to fit with the dominating paradigm. Consciousness may there be considered an emergent phenomenon, but with no causative power on the underlying neural processes, which would require downward causation. In traditional, reductionist science, such downward causation is difficult to accept. Moreover, neither randomness nor deterministic laws of nature, which are the only scientific explanations available for what happens in the world, seem to allow for free will, as mentioned in the Introduction.

On the other hand, CCW seems to fit better with our experience and our social/cultural traditions, where responsibility for our actions appears to require a capacity to consciously choose between alternative actions. So, is there a way out of this dilemma? Could there be any room for free will in current science, after all? I concur that downward causation is necessary but not sufficient for demonstrating the existence of free will. However, in order to demonstrate downward causation, one has to show that a change in some high level variable(s) reliably results in a change in lower-level variable(s).

For a nervous system, as for complex systems in general, different phenomena appear at different levels of aggregation. Emergent phenomena may result from an upward, “bottom-up” causation, based on *micro* level phenomena. Yet, higher *macro* levels may also “control” lower ones (c.f. the so-called enslaving principle of Haken 1983), an example of downward, “top-down” causation. This interplay between micro and macro levels is part of what frames the dynamics of neural systems, and is another example of *circular causality,* which Freeman referred to for the action-perception cycle (Freeman 1999; see also the section on intentionality above). Of special interest is the *meso* level, i.e., the level in between the micro and the macro, where bottom-up meets top-down.

Mesoscopic brain dynamics is partly a result of a dynamic balance between opposing processes such as inhibition and excitation, which often results in oscillatory and chaotic-like behaviour (Freeman 2000; Liljenström 2012). Yet, this dynamics is mixed with noise, generated at a microscopic level by spontaneous neural activity. It is also affected by macroscopic activity, such as slow rhythms generated by cortico-thalamic circuits or neuromodulation from different brain regions. Effects of arousal, attention, or mood, through neuromodulation or other means, could be seen as a top-down interaction from macroscopic activity to mesoscopic neurodynamics. Our computational models have demonstrated how complex neurodynamics of cortical networks can influence the neural activity of single or populations of neurons. We have also shown how neuromodulation and attention can synchronize and in other ways regulate the activity of neurons and neural populations in networks (Liljenström 2016).

While the behavior of single molecules or cells may appear stochastic and noisy at micro- and mesoscopic levels, their collective behavior could generally be regarded as deterministic and ordered at the *macroscopic* level, where the irregularities at lower levels are “averaged out”. Still, under certain circumstances, single events at these lower levels may be amplified and cause state transitions or other phenomena at macroscopic levels. For example, retinal absorption of single photons under extremely dark conditions, or odor receptor absorptions of single odorous molecules can be amplified by various neural networks to result in conscious perception.

In addition, the complex neurodynamics of cortical networks may result in unpredictable chaotic behavior with a high sensitivity to “initial conditions”, so very small differences in cortical firing patterns may change the activity at cortical network levels and result in completely different output signals. Both experimental studies (Freeman 2000) and computer simulations (Liljenström 1995) indicate that the complex neurodynamics of cortical systems can provide chaotic intentional states, which eventually may converge to meaningful percepts. Mental processes may arise from neural activity, but they may also affect the neural activity, in a kind of downward causation that seems necessary for free will. The intricate web of inter-relationships between processes at different organizational levels of neural systems seems able to provide both upward and downward causation.

All of this might provide possibilities for several optional outcomes, “choices” of any brain-state. It may constitute necessary, but not sufficient conditions for free will. There also needs to be “someone”, a subject, to make the choices, in order for free will to exist. The subject may well be formed by the global activity of the brain-mind system, under constraints given by the physical structures, but it should be in control of its actions.

Indeed, the main reason why science has problems encompassing free will is that it seems to require the action of a conscious agent, and (natural) science has so far only dealt with objects, not agents/subjects. The theories and laws of physics were developed for inanimate, comparatively simple objects and their interaction, and have very little to say about the behavior of complex biological systems, in particular regarding brain-mind systems and their (inter-)actions. In fact, Einstein, as well as Schrödinger (1944) recognized the insufficiency of contemporary physics to describe the immense complexity of living systems. In lack of conclusive experimental evidence, perhaps theoretical insights, such as Einstein’s of space and time as non-separate and interdependent, could consider mind and matter as non-separable, and consciousness and agency as an essential part of our natural world. Indeed, when intentional or conscious actions include also the choice of mating partners, the caring for offsprings, and relational behavior in general, it is clear that consciousness may not only have effects at an individual level, but can also be seen as a driving force in evolution.

To conclude, we are embedded in this world in an ongoing action-perception cycle, continuously interacting with our environment. Causal relationships are difficult to determine, and external and internal influences (preferences, expectations, intentions, emotions) affect our unconscious and conscious actions. This is particularly obvious in a social context, in our interaction with others. However, if we should be accounted responsible for our actions, it appears obvious that we must be able to choose between different actions, that we must have a free will. Until we have more evidence to make any scientific conclusions about the existence of free will, it may be wise to be humble and rather rely on our intuition than on any counter-intuitive hypothesis that seems reasonable, just because it fits with the current paradigm. In order to allow for consciousness and free will, science probably needs to be extended beyond chance and necessity, which currently are its only models of explanation.
